# Intranasal cerium oxide nanoparticles improves locomotor activity and reduces oxidative stress and neuroinflammation in haloperidol-induced parkinsonism in rats

**DOI:** 10.3389/fphar.2023.1188470

**Published:** 2023-05-30

**Authors:** Mohammad, Urooj Ahmed Khan, Musarrat Husain Warsi, Huda Mohammed Alkreathy, Shahid Karim, Gaurav Kumar Jain, Asgar Ali

**Affiliations:** ^1^ Department of Pharmaceutics, School of Pharmaceutical Education and Research, New Delhi, India; ^2^ Department of Pharmaceutics, Dr. Ram Manohar Lohia College of Pharmacy, Ghaziabad, Uttar Pradesh, India; ^3^ Department of Pharmaceutics and Industrial Pharmacy, College of Pharmacy, Taif University, Taif, Saudi Arabia; ^4^ Department of Pharmacology, Faculty of Medicine, King Abdulaziz University, Jeddah, Saudi Arabia; ^5^ Department of Pharmaceutics, Delhi Pharmaceutical Sciences and Research University, New Delhi, India; ^6^ Center for Advanced Formulation Technology, Delhi Pharmaceutical Sciences and Research University, New Delhi, India

**Keywords:** Parkinson disease, brain targeting, antioxidant activity, gamma scintigraphy, inorganic nanoparticles

## Abstract

**Introduction:** Cerium oxide nanoparticles (CONPs) have been investigated for their therapeutic potential in Parkinson’s disease (PD) due to their potent and regenerative antioxidant activity. In the present study, CONPs were used to ameliorate the oxidative stress caused by free radicals in haloperidol-induced PD in rats following intranasal administration.

**Method:** The antioxidant potential of the CONPs was evaluated *in vitro* using ferric reducing antioxidant power (FRAP) assay. The penetration and local toxicity of the CONPs was evaluated *ex-vivo* using goat nasal mucosa. The acute local toxicity of intranasal CONPs was also studied in rat. Gamma scintigraphy was used to assess the targeted brain delivery of CONPs. Acute toxicity studies were performed in rats to demonstrate safety of intranasal CONPs. Further, open field test, pole test, biochemical estimations and brain histopathology was performed to evaluate efficacy of intranasal CONPs in haloperidol-induced PD rat model.

**Results:** The FRAP assay revealed highest antioxidant activity of prepared CONPs at a concentration of 25 μg/mL. Confocal microscopy showed deep and homogenous distribution of CONPs in the goat nasal mucus layers. No signs of irritation or injury were seen in goat nasal membrane when treated with optimized CONPs. Scintigraphy studies in rats showed targeted brain delivery of intranasal CONPs and acute toxicity study demonstrated safety. The results of open field and pole test showed highly significant (*p* < 0.001) improvement in locomotor activity of rats treated with intranasal CONPs compared to untreated rats. Further, brain histopathology of treatment group rats showed reduced neurodegeneration with presence of more live cells. The amount of thiobarbituric acid reactive substances (TBARS) was reduced significantly, whereas the levels of catalase (CAT), superoxide dismutase (SOD), and GSH were increased significantly, while amounts of interleukin-6 (IL-6) and tumour necrosis factor-alpha (TNF-α) showed significant reduction after intranasal administration of CONPs. Also, the intranasal CONPs, significantly high (*p* < 0.001) dopamine concentration (13.93 ± 0.85 ng/mg protein) as compared to haloperidol-induced control rats (5.76 ± 0.70 ng/mg protein).

**Conclusion:** The overall results concluded that the intranasal CONPs could be safe and effective therapeutics for the management of PD.

## 1 Introduction

Parkinson’s disease (PD) is a neurological condition that worsens over time as a result of the loss of a small number of neurons that control movement and characterized by postural instability, bradykinesia, rigidity, and tremor (Bloem et al., 2021). According to recent reports, PD affects nearly 10% of adults over the age of 60 and an estimated 10 million people worldwide (or roughly 0.3% of the world total population) (Surathi et al., 2016; Ray Dorsey et al., 2018). In PD, the substantia nigra region of the brain has a dopamine depletion that results in unpredictable motor movements and a number of non-motor symptoms (Kalia and Lang, 2015; Rajan et al., 2020). One of the primary factors that leads to cellular malfunction and neuronal cell death in PD is oxidative stress (Pangeni et al., 2014). All the body tissues continuously produce free radicals and reactive oxygen species (ROS). However, oxidative stress develops when the activity of cellular antioxidants is out of balance with ROS generation (Pizzino et al., 2017). The basic theory underlying the degeneration of dopaminergic neurons linked with PD is the concept of free radical-mediated neuronal damage (Iarkov et al., 2020). The brain is more vulnerable to ROS imbalance because of its high oxygen demand and abundance of polyunsaturated fatty acids, proteins, nucleic acids, and lipids that are prone to oxidation (Elnaggar et al., 2015). Additionally, number of neuroinflammatory processes may trigger oxidative stress conditions that result in neural cell death (Blesa et al., 2015). Standard antioxidants have been used to reduce the degenerative alterations in PD albeit with little effect (Chang and Chen, 2020; Duarte-Jurado et al., 2021).

Recently, nanoparticles (NPs) have been widely utilized for variety of brain disorders. NPs demonstrated target delivery to brain, enhanced bioavailability, protection of drug from degradation, reduction of side-effects and dose reduction (Silva et al., 2021). Usually, NPs are used as drug carriers or vehicles, but several inorganic NPs such as those of gold, silver, zinc and ceramic oxide have been used as therapeutic agents (Wen et al., 2016; Din et al., 2017; Shaabani et al., 2017; Vilella et al., 2018; Ruotolo et al., 2020). Among inorganic NPs, CONPs have potential for management of variety of brain disorders owing to their strong antioxidant potential (Rzigalinski et al., 2017).

CONPs unique characteristic to transit between oxidation states (Ce^3+^/Ce^4+^) and to bind oxygen irreversibly allows effective ROS scavenging (Dhall and Self, 2018). Additionally, CONPs display catalase and superoxide dismutase mimicking capabilities (Song et al., 2021) and are therefore expected to be more effective than other antioxidants at scavenging practically all types of ROS. Unlike conventional antioxidants, CONPs radical scavenging ability is regenerable, allowing for prolonged activity (Heckert et al., 2008).

Numerous studies demonstrated that CONPs shield neurons and other cells including neurons against ROS-induced damage (Schubert et al., 2006; Niu et al., 2011; Hegazy et al., 2017; Kong et al., 2011). In regards to neurological conditions, CONPs demonstrated promising therapeutic potential in several neurodegenerative disorders, like Pakinson’s disease, alzheimer’s disease, traumatic brain injury and multiple sclerosis, where oxidative stress plays a significant role (D’Angelo et al., 2009; Sandhir et al., 2015; Hegazy et al., 2017; Ruotolo et al., 2020).

There are several animal models explained in literature used to induce PD experimentally in rats or mice. Traditionally, 1-methyl-4-phenyl-1,2,3,6-tetrahydropyridine (MPTP), 6-hydroxydopamine (6-OHDA)-induced neurotoxic models and agrochemicals including the insecticide rotenone, the herbicide paraquat, and the fungicide maneb as part of neurotoxin-based techniques are used. However, there is no animal disease model that completely mimics human PD because of its complicated pathology (Briñez-Gallego et al., 2023). Haloperidol-induced catalepsy is frequently utilised as a mouse model for the study of motor impairments seen in PD and for the screening of prospective anti-parkinsonism compounds (Waku et al., 2021). Thus, haloperidol-induced PD in rats was used to evaluate efficacy of CONPs in the present work.

Among the biggest difficulties in neurological conditions is to formulate an efficient therapeutic strategy or approach that can successfully cross the blood brain barrier (BBB) (Saraiva et al., 2016; Md et al., 2018). A potentially effective method of delivering medications to the brain is through the nasal mucosa, trigeminal neurons, and olfactory pathways (Johnson et al., 2010; Pardeshi and Belgamwar, 2013; Jeong et al., 2023). Drugs with limited oral bioavailability or those that go through first pass metabolism may nevertheless be able to reach the brain when administered intranasally (Pardeshi and Belgamwar, 2013). Because it targets the brain directly, intranasal delivery reduces the likelihood of side effects and avoids intrusive systemic administration (Keller et al., 2022). Researchers frequently use the intranasal method to treat neurodegeneration because it is important for bypassing the BBB and delivering drugs directly to the brain (Bahadur et al., 2020; Su et al., 2020). Furthermore, the results demonstrated that NPs immediately enter into the CSF by subarachnoid absorption by the nasal epithelium (Bonferoni et al., 2020). Recently, intranasal CONPs prepared in our lab have shown therapeutic potential for the management of AD (Danish et al., 2022). Though, the efficacy of CONPs in PD is extensively reported, we hypothesized that intranasal administration might result in targeted brain delivery leading to reduced dose, improved efficacy and reduced side-effects. Thus, intranasal CONPs were evaluated *in vitro* and *in vivo*.

The antioxidant potential of the CONPs was evaluated *in vitro* using ferric reducing antioxidant power (FRAP) assay. The penetration and local toxicity of the CONPs was evaluated *ex-vivo* using goat nasal mucosa. The acute local toxicity of intranasal CONPs was also studied in rat. Gamma scintigraphy was used to assess the *in vivo* nose to brain delivery of CONPs following radiolabeling of CONPs with technetium-99m. *In vivo* motor manifestation and biochemical estimation was performed to evaluate efficacy of CONPs in haloperidol-induced PD rat model.

## 2 Materials and methods

### 2.1 Materials

Cerium nitrate hexahydrate (Ce(NO_3_)_3_.6H2O, 99.9%), ammonia, haloperidol, levodopa, and FRAP were procured from Sigma Aldrich (India). TNF-α and IL-6 ELISA kits were purchased from Krishgen Biosystems (India). AR grade methanol was purchased from Merck (India) whereas acetonitrile AR grade was obtained from Thomas Baker (India). 5,5′-dithio-bis-2-nitrobenzoic acid (DTNB), Rhodamine-123, and dopamine HCl were bought from Sigma-Aldrich (India). Ammonium acetate and acetonitrile of MS grade were acquired from Merck (India). All other chemicals and reagents were of analytical grade.

### 2.2 Development and characterization of CONPs

CONPs were prepared by modification of previously published homogenous precipitation method (Danish et al., 2022). Briefly, tween 80 was dissolved in methanol: water solution (20 mL) and to this cerium (III) nitrate hexahydrate was added. The mixture was kept at 50°C and to this mixture ammonia (20 mL, 3 M) was added. The reaction was then allowed to run for 2 hours with constant stirring at 500 rpm. The yellow suspension, so produced was centrifuged for 5 minutes at 2000 rpm followed by two washes with a methanol: water solution to produce CONPs. The CONPs were dried overnight at 50°C and stored in refrigerator at 4°C until used. The CONPs were characterized for size by dynamic light scattering, for morphology by transmission electron microscopy and for stability by zeta potential.

### 2.3 Ferric reducing antioxidant power (FRAP) assay

The FRAP assay was carried out in accordance with the procedure illustrated by Benzie and Strain (1996), Benzie and Strain (1996). The antioxidant activity in the FRAP assay was stated in terms of mM Fe^2+^, and it expressed the corresponding amount of antioxidant needed to reduce the Fe^3+^to Fe^2+^ions in the Fe^3+^ -TPTZ complex. The FRAP mixture was made by mixing 10 mM of 2,4,6-tripyridyl-s-triazine (TPTZ), 300 mM acetate buffer (3.1 g sodium acetate trihydrate and 16 mL acetic acid, pH 3.6) and 20 mM ferric chloride hexahydrate in 40 mM HCl. The working standard was made by combining 5 mL of TPTZ solution with 5 mL of ferric chloride hexahydrate in 50 mL of acetate buffer and heating up to 37°C. CONPs (100 µL) with a concentration of 5–25 μg/mL, were added to 4 mL of FRAP reagent and collected in various test tubes. Sample and FRAP solution were combined, and the reaction was allowed to occur for 30 min in the dark. Absorbance values for the coloured substances (ferrous tripyridyl triazine complex) were recorded by UV-vis spectrophotometer at 593 nm.

### 2.4 *Ex-vivo* nasal mucosa penetration study

**FIGURE 1:**
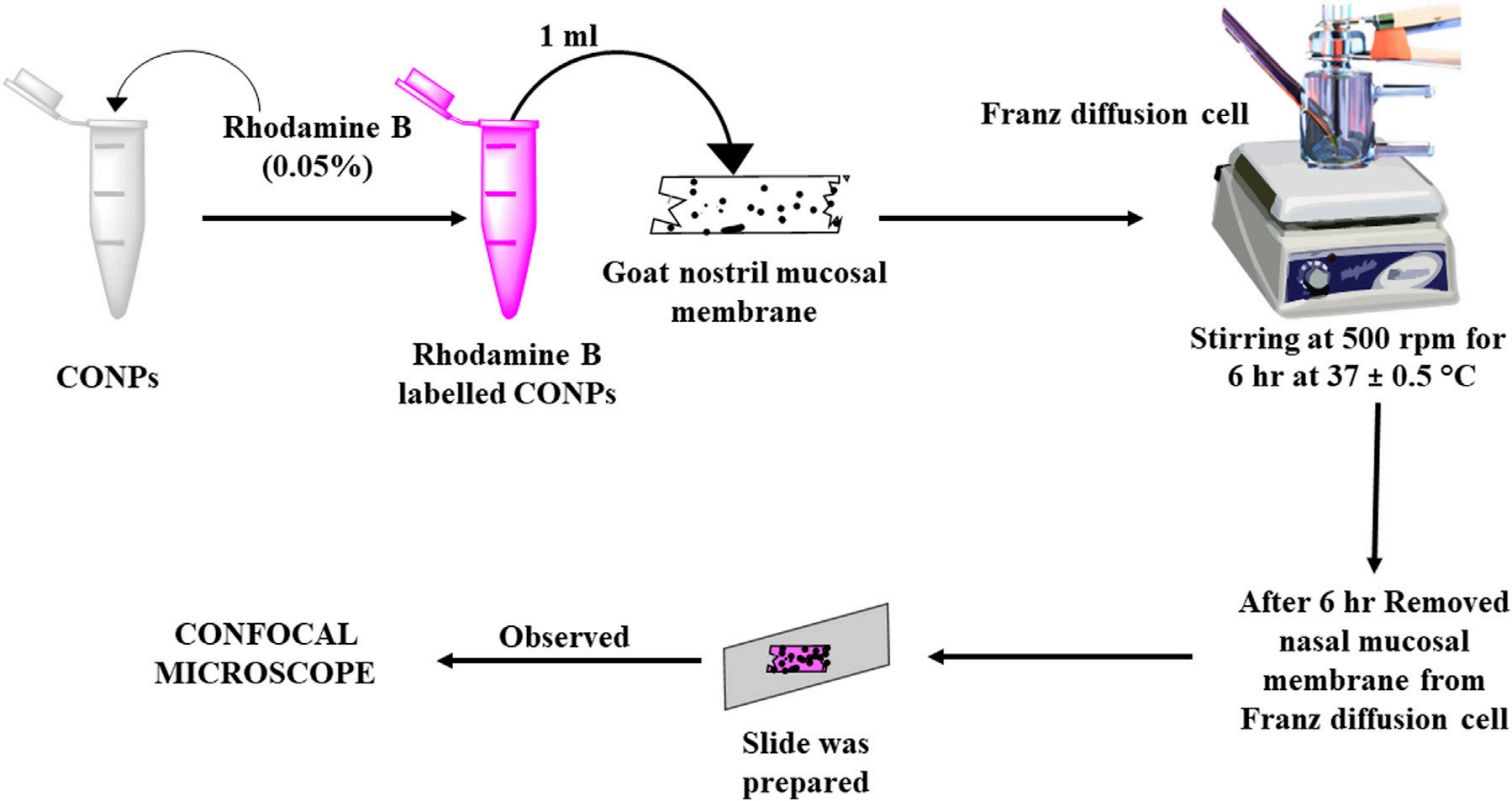
Schematic diagram showing study plan for assessment of depth of penetration using CLSM (Confocal Laser Scanning Microscopy).

*Ex-Vivo* nasal mucosa penetration study was conducted via confocal microscopy for assessing the penetration of the synthesized CONPs to nasal mucosal membrane using the dye rhodamine B with emission and excretion energy at 625 nm and 540 respectively (Gao, 2016). Rhodamine B dye (0.05%) was incorporated into CONPs by covalently modifying the surface with amino groups. This is achieved by treating the surface OH groups with 3-aminopropyltriethoxysilane in dry toluene, resulting in the formation of NH_2_-CONPs. The reaction occurs under neutral conditions and creates strong Ce-O-Si bonds between the amino group and the CONPs. These amino groups serve as tethers that allow RhB to attach to the CONPs. The next step involves reacting the aminated NH_2_-CONPs with RhB isothiocyanate, resulting in a strong thiourea linkage between the organic fluorophore and the inorganic CONPs. After this, 1 mL of produced rhodamine B labelled CONPs was added onto freshly removed goat nasal mucosal membrane mounted on a Franz diffusion cell for 6 hours using phosphate buffer solution (pH 6.4) as the diffusing medium (Figure 1). This cell was placed on a continuous stirring and allowed to agitate extremely slowly while the media temperature was held at 37°C ± 0.5°C. Stirring was permitted until the colour had barely diffused throughout the medium (around 6 hours). The nasal mucosal membrane was removed from Franz diffusion cell, a slide was created and observed under confocal microscope (Leica TCS SP8, Zeiss, Germany).

### 2.5 *Ex vivo* nasal cilio toxicity study

**FIGURE 2:**
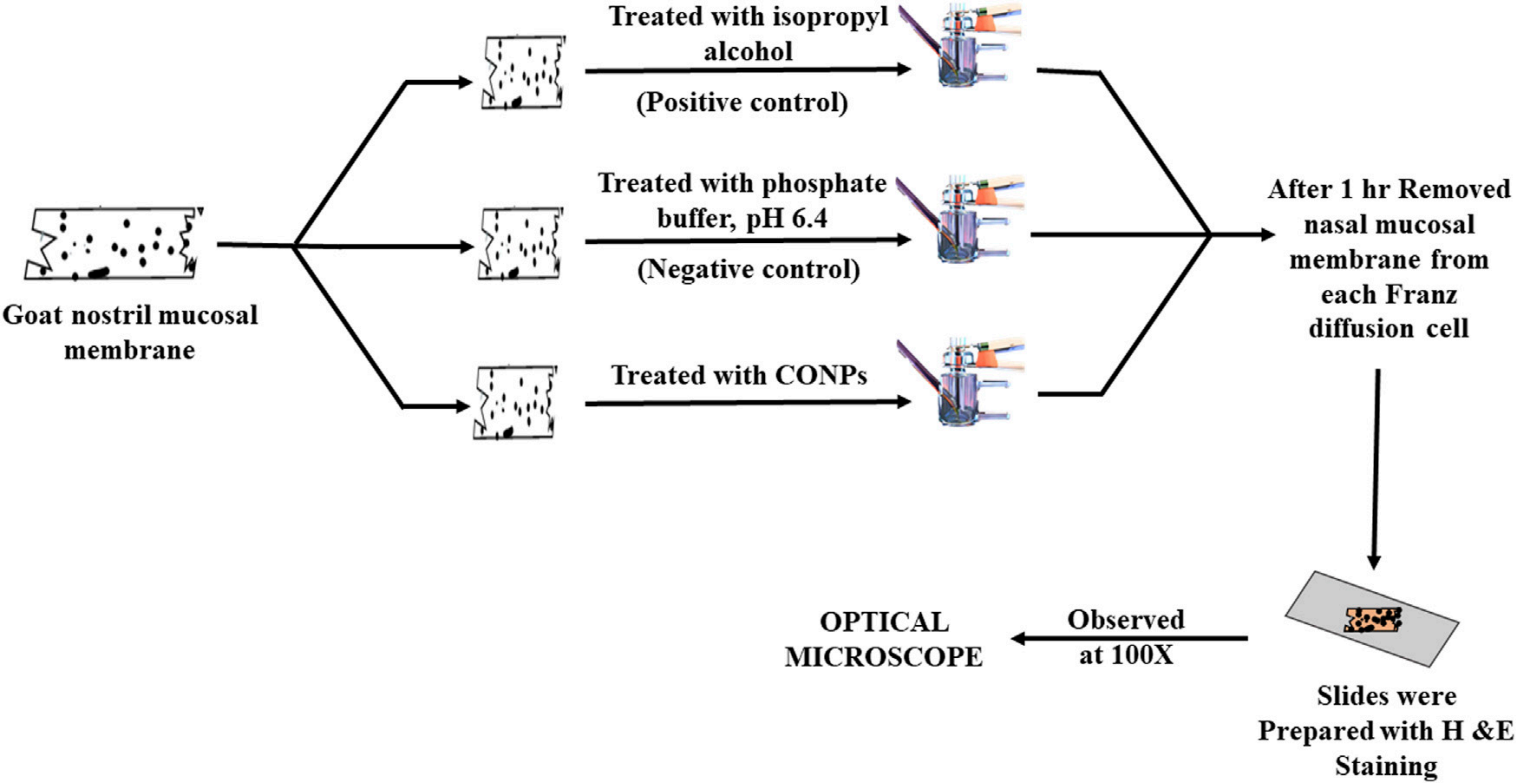
Schematic diagram showing study plan for nasal cilio toxicity study.

The nasal cilio toxicity of the CONPs, was assessed using goat nasal mucosa. The goat nasal mucosa was divided into three sections of equal thickness, each of which was mounted on separate Franz diffusion cells. The three sections were treated separately with CONPs, isopropyl alcohol (positive control), and phosphate buffer, pH 6.4 (negative control) for 1 h (Figure 2). Following this, sections were collected, stained with haematoxylin and eosin and analysed for histopathological changes using microscope at 100× magnification. (Usama Ashhar et al., 2022).

### 2.6 Targeted brain delivery of intranasal CONPs by scintigraphy

#### 2.6.1 Radiolabeling of CONPs with 99mTc

Sodium pertechnetate, freshly eluted from a ^99^Mo/^99m^Tc generator was used for radiolabeling in accordance with standard protocol of Institute of Nuclear Medicine and Allied Sciences (INMAS). Briefly, 2 mg/mL stannous chloride (reducing agent) was mixed with 50 mg of CONPs powder and the prepared mixture was dried at 50°C in an oven. Then, 10 µL of 99mTc was added into the mixture and mixture was dried in oven to get radiolabeled CONPs. Before delivering the radiolabeled compound to animals for scintigraphy investigations, the labeling efficacy was assessed using instantaneous thin layer chromatography (ITLC) in both *in vivo* and *in vitro* media using following equation (Eq. 1) (Khan et al., 2020).

$$Radiolabeling\;efficiency=\frac{Counts\;on\;bottom\times 100}{Counts\;on\;top+bottom}$$ (1)

#### 2.6.2 Gamma scintigraphy investigation

**FIGURE 3:**
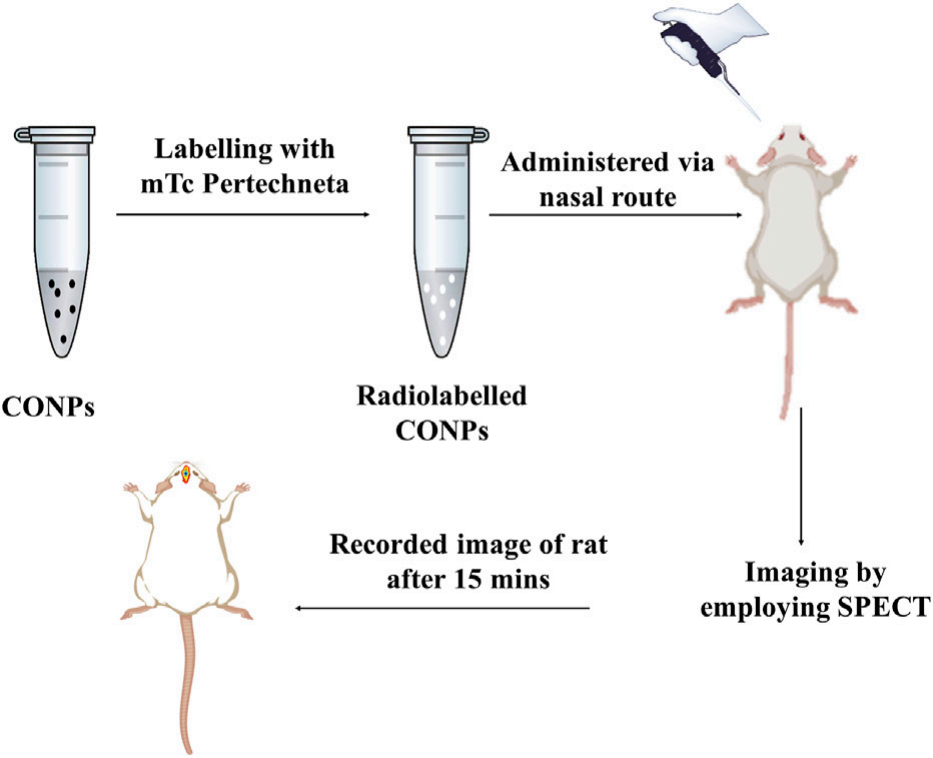
Graphic representation of a SPECT scans of rats to validate drug accumulation and disposal in the brain.

The gamma scintigraphy (Figure 3) was used to confirm the brain targeting of intranasal CONPs. Considering proof-of-concept study, only three healthy male Sprague Dawley rats of weight approx. 250 ± 30 g, aged 6–7 months were used. Twenty four hours before of the experiment, rats remained in a fasted state. Further, radiolabeled intranasal CONPs (50 µCi of ^99m^Tc) were administered to rats using micro pipette. To understand the brain delivery of CONPs, the images were obtained at 15, 30 and 60 min using gamma camera (Siemens T2 SPECT-CT).

### 2.7 Safety (acute toxicity) study

Six adult, male, Wistar rats weighed 250 ± 30 g, aged 6–7 months were used for acute toxicity study. Acute toxicity was carried out for 14 days and the change in weight of each rat was observed (Ekanayake et al., 2019). The rats were divided into two groups (*n* = 3). Group I received normal saline whereas group II was given intranasal CONPs (6 mg/kg, 10 µL) for 14 days. At day 14, percent change in body weight was calculated by using Eq. 2.

$$Percent\;change\;in\;body\;weight=\frac{Body\;weight\;at\;termination\;ofstudy-Initial\;body\;weight}{Initial\;weight}X\;100$$ (2)

### 2.8 Efficacy Study

Sixteen adult, male, Wistar rats weighed 235 ± 15 g, aged 5–6 months were used for efficacy study. The rats were divided into four groups, having 4 rats each (*n* = 4). Group 1 consisted of healthy rats treated with intranasal normal saline (10 µL) for 14 days and considered as negative control. Group 2, i.e., haloperidol-induced control group, received intraperitoneal haloperidol (0.23 mg/kg) for 14 days. Group 3 received oral levodopa suspension (4.97 mg/kg) + intraperitoneal haloperidol (0.23 mg/kg) for 14 days and considered as haloperidol-induced treated with the SD drug. Further, group 4 received intranasal CONPs (6 mg/kg) + oral levodopa suspension (4.97 mg/kg) along with intraperitoneal haloperidol (0.23 mg/kg) and considered as haloperidol-induced treated with CONPs. Where ever used, haloperidol was administered for 30 min post-treatment. Normal saline and levodopa suspension was given continuously for 14 days whereas CONPs were given on day 0 and day 7. The behavioural and locomotor assessment through open field test and pole test were conducted. Thereafter, histopathology of brain cells was performed in 1 animal from each group to study neuronal damage and degeneration. The brain of remaining 3 animals in each group was used for biochemical estimation to study changes in oxidative stress and neuroinflammation.

### 2.9 Behavioural studies

#### 2.9.1 Open field test

The open field test is a behavioural technique used to assess motor activity as a reaction to an unknown environment. The purpose of this test was to evaluate rearing and impulsive locomotor activities (Feyissa et al., 2017). Rats were put in a corner square (50 cm × 30 cm × 20 cm) of an open field arena and watched for 3 min. A count was made of the number of squares entered (all 4 paws within the square), as well as the number of rears. The latency to rear and move were also measured (Hegazy et al., 2017).

#### 2.9.2 Pole test

The pole test is used to examine impairment in exploratory behaviour and motor manifestations. This assessment evaluates bradykinesia. It is typically used to assess movement difficulties in animals that are associated to the ganglia. Rats were positioned head-up towards the top of a vertical, 55 cm-tall wooden poles with 1 cm diameter. Usually, after being placed on the pole, the rat settles itself downwards, descends the pole and then returns to the cage. Both the time spent turning in a downward direction (t-turn) and the overall amount of time spent moving down (t-total) were observed (Meredith and Kang, 2006).

### 2.10 Histopathological study

#### 2.10.1 Preparation of tissue samples

The brain of one rat from each group were removed following neurobehavioral tests, cleansed with ice-cold saline at room temperature (RT), and then preserved in a 10% formaldehyde solution at RT, and finally embedding in paraffin after isolation. Utilizing xylene and ethanol, pieces were deparaffinized after being cut at a thickness of 5 µm. Haematoxylin and eosin were used to stain the sections completely.

#### 2.10.2 Histopathological analysis

Histopathological analysis was performed on all the groups with haloperidol-induced PD to evaluate brain segments using haematoxylin and eosin staining. The investigation’s objective was to assess the neuronal damages caused by haloperidol and the contribution of CONPs in minimising the damages. The slides prepared above were inspected through a microscope with a 100× magnification (Hasan et al., 2021).

### 2.11 Biochemical estimations

#### 2.11.1 Preparation of brain homogenate

The brains were removed after anesthetizing rats (*n* = 3) on 15th day, cleaned and rinsed with ice cooled saline solution at room temperature. Homogenization was done in an ice bath for preventing overheating using 0.1 M phosphate buffer (10 times the weight of tissue) having pH 7.4. The homogenate was allowed to dissociate completely in the buffer by placing it on ice for five to 10 minutes. Centrifugation was then performed at 4°C for 20 min using 10,000 rpm. The supernatant so obtained was collected and stored at –80°C till further used.

#### 2.11.2 Estimation of biomarkers

ROS are the main sources of oxidative stress and their formation is stimulated by haloperidol inside the brain (Adedeji et al., 2014). Biochemical assessments were performed at Nanoformulation lab with the help of Central instrumentation facility, Jamia Hamdard, to estimate the levels of superoxide dismutase (SOD), GSH, catalase (CAT), Thiobarbituric substances (TBARS), and GSH in all the above-mentioned animal groups. Along with oxidative stress measurements, levels of neuroinflammation cytokines like tumour necrosis factor-alpha (TNF-α) and interleukin-6 (IL-6) were also estimated. The prepared brain homogenate was used to estimate biomarkers of oxidative stress along with IL-6 and TNF-α (Frijhoff et al., 2015).

#### 2.11.3 TBARS estimation

The TBARS level is frequently used to evaluate lipid peroxidation. The estimation of TBARS content was done using the method published in literature with slight modifications (Kumar et al., 2018). A test tube containing 0.2 mL of brain homogenate, about 9 mL KCl (1.15%), 0.2 mL sodium dodecyl sulphate (8.1%), and 1.5 mL acetic acid (20%) were poured. The pH of the solution was adjusted to 3.5 using NaOH solution. Then, 1.5 mL aqueous solution of thiobarbituric acid (0.8% w/v) was added. The produced mixture was heated to 80°C for 60 min before being cooled. Further n-butanol and pyridine mixture (5 mL) in the ratio of 30:2 w/v respectively was added in the above solution along with 1 mL of distilled water and then centrifuged the mixture for 15 min at 4,500 rpm. The organic layer’s absorbance was recorded against blank at 532 nm. The content of TBARS was determined as nmol/mg of protein by using a 1.56 × 10^−5^ cm nmol^-1^ molar extinction coefficient.

#### 2.11.4 GSH estimation

A test tube carrying 2.5 mL of 0.02 M EDTA and 500 mg of brain were filled with the contents after being weighed, homogenised utilising the tissue homogenizer, and then topped off with 0.02 M EDTA to the point of 5 mL trichloroacetic acid, 50 percent, and water, 4 mL, were combined with homogenate, 5 mL. (TCA). Following a 10–15-min period of intermittent shaking, samples were centrifuged for 15 min at 3,000 rpm. Then, 4 mL of 0.4 M Tris buffer (pH 8.9) were added into 2 mL of supernatant obtained from above procedure. After that, 0.01 M 5, 5′-dithiobis-(2-nitrobenzoic acid) (0.1 mL) was added into the above mixture. A mixture of chemicals was used as a blank reference (i.e., 1 mL of 50 percent TCA, 4 mL distilled water, 0.01 M DTNB and 4 mL of 0.4 M Tris buffer) and absorbance was recorded at 412 nm. The equation below was used to determine how much GSH was present in the tissue (Gupta et al., 2018).

$$GSH\;\left(µmol\;/\;mg\;of\;protein\right)=\frac{Absorbance\;at\;412\;nm}{E\;X\;mg\;of\;protein}\;X\;D$$ (3)

Where, D and E are dilution factor and DTNB’s extinction coefficient, respectively.

#### 2.11.5 SOD estimation

In this experiment, potassium phosphate buffer (2 mL) was initially combined with 200 mg of cerebrum/brain homogenate (pH 7.4). The resulting mixture was centrifuged at 1,000 rpm for 10 min at 4°C.100 μL of the supernatant solution was mixed with 25 μL of pyrogallol and 3 mL of Tris HCL buffer. The absorbance of the obtained mixture was determined at 420 nm. SOD concentration was estimated as mol/mg of protein (Sharma et al., 2020).

#### 2.11.6 CAT estimation

The CAT levels were estimated using the method described by Rajan and others (Kumar et al., 2020). A final amount of 3 mL was obtained by mixing 0.05 mL of supernatant with 1.95 mL of 0.5 M phosphate buffer (pH 7.4) in 0.019 M H_2_O_2_ (hydrogen peroxide). The resultant solution’s absorbance was recorded at 240 nm. The quantity of CAT was determined as nmol of hydrogen peroxide per minute per mg of protein.

#### 2.11.7 TNF-α and IL-6 estimation via ELISA

The TNF-α and IL-6 (neuro - inflammatory cytokines) were quantified using specific ELISA kits obtained from Krishgen Biosystems. The ELISA kits were based on the principle of sandwich techniques, and the assessments were executed in accordance with the instructions provided for the specific cytokine kit. The pre-coated antibody in the wells was exposed to the standard and samples where they allowed get adhered. The plate was then incubated as advised after being washed with buffer to remove unbound materials. A suitable amount of washing was followed by the addition of an enzyme-linked polyclonal antibody specific to a particular inflammatory cytokine into the wells and incubated. The unbound antibodies from samples were removed after washing. Each well received an adequate amount of substrate solution, which produces a blue colour that turns yellow when a stop solution is added. At last, the absorbance was recorded at 450 nm using ELISA reader (Zameer et al., 2020).

### 2.12 Dopamine assay using RP-HPLC method (modified)

0.2 M HClO_4_ containing 100 M EDTA disodium salt was used to homogenise a portion of the striatum (20% w/v) for determining dopamine concentration. For 30 min, the homogenate was allowed to deproteinize. The homogenate was then centrifuged for 15 min at 10,000 rpm at 0°C (MX-305, Tomy, Japan). The pH of the supernatant was adjusted with 1 M acetic acid to pH = 3.5 and 0.45 µm membrane filter was used for filtration (Millipore, United States). The reversed–phase HPLC analytical method explained by Arsene AL and co-workers for the determination of dopamine was adopted with some modifications (Arsene et al., 2009). Briefly, 5 µm RP 18 (C18) column, Lichrospher^®^100 (250 mm × 4.6 mm) attached to an HPLC (Shimadzu) equipped with variable wavelength programmable UV/VIS detector, and a Rheodyne injector with a 20 µL loop was used. For the drug analysis class-VP 5.032 software was used. 0.8 mM EDTA disodium salt, 0.65 g sodium heptane sulphonate, 0.12 M sodium dihydrogen phosphate, and 65% methanol was used as mobile phase. Amount of Dopamine was determined at 210 nm using UV detector. HPLC results were expressed as ng/mg protein after adjusting to total tissue proteins.

### 2.13 Animals

Rats were housed in polypropylene cages under the consistent temperature (25°C ± 2°C), humidity (50%–60%), and 12 h light-dark environments and supplied with standard laboratory diet and water ad libitum. The protocol (173/CPCSEA/2000; Approval No. 1611,2019) was approved by the Institutional Animal Ethical Committee of the Jamia Hamdard and all the tests were conducted in accordance with laboratory animal ethical guidelines.

### 2.14 Statistical analysis

One-way ANOVA followed by *post hoc* Tukey’s test was performed for all the statistical investigations using the IBM SPSS Statistics, Version 20.0. OriginPro 2016 software (Origin Lab Corporation, Northampton, MA, United States) was used to plot the graph for the results of the experiments. All of the studies were done in triplicate, and the data were provided as mean ± standard deviation.

## 3 Results

### 3.1 Development and characterization of CONPs

CONPs were prepared by modified precipitation method using tween 80 as stabilizer and methanol/water as solvent. The CONPs were small-sized (105.1 ± 5.78 nm), spherical (TEM), uniform (PDI, 0.119 ± 0.006) and stable (Zeta potential, −22.7 ± 1.03 mV).

### 3.2 Ferric reducing antioxidant power (FRAP) assay

**FIGURE 4:**
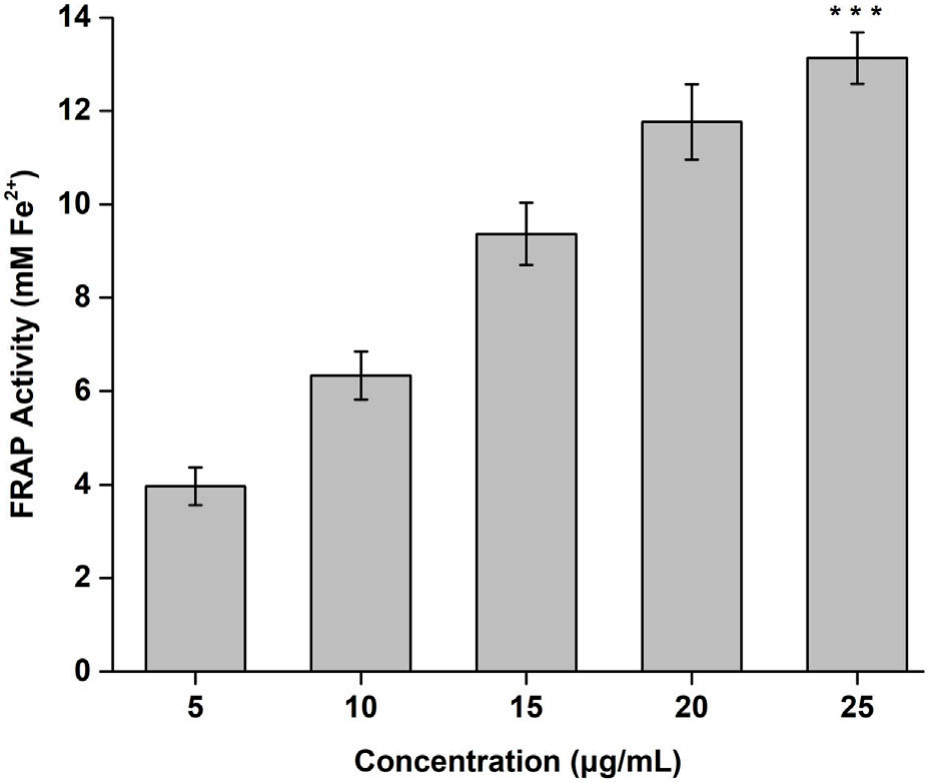
FRAP antioxidant activity of CONPs. The significance was determined as ***p < 0.001 *versus* FRAP activity at 5 μg/mL.

The FRAP activity of the CONPs determined in concentration range of 5–25 μg/mL was shown in Figure 4. Highest FRAP activity (13.13 mM) was observed for CONPs at the concentration of 25 μg/mL. The results also demonstrated antioxidant potential of CONPs and their ability to reduce Fe^3+^ - TPTZ complex to Fe^2+^.

### 3.3 *Ex vivo* nasal mucosa penetration study

**FIGURE 5:**
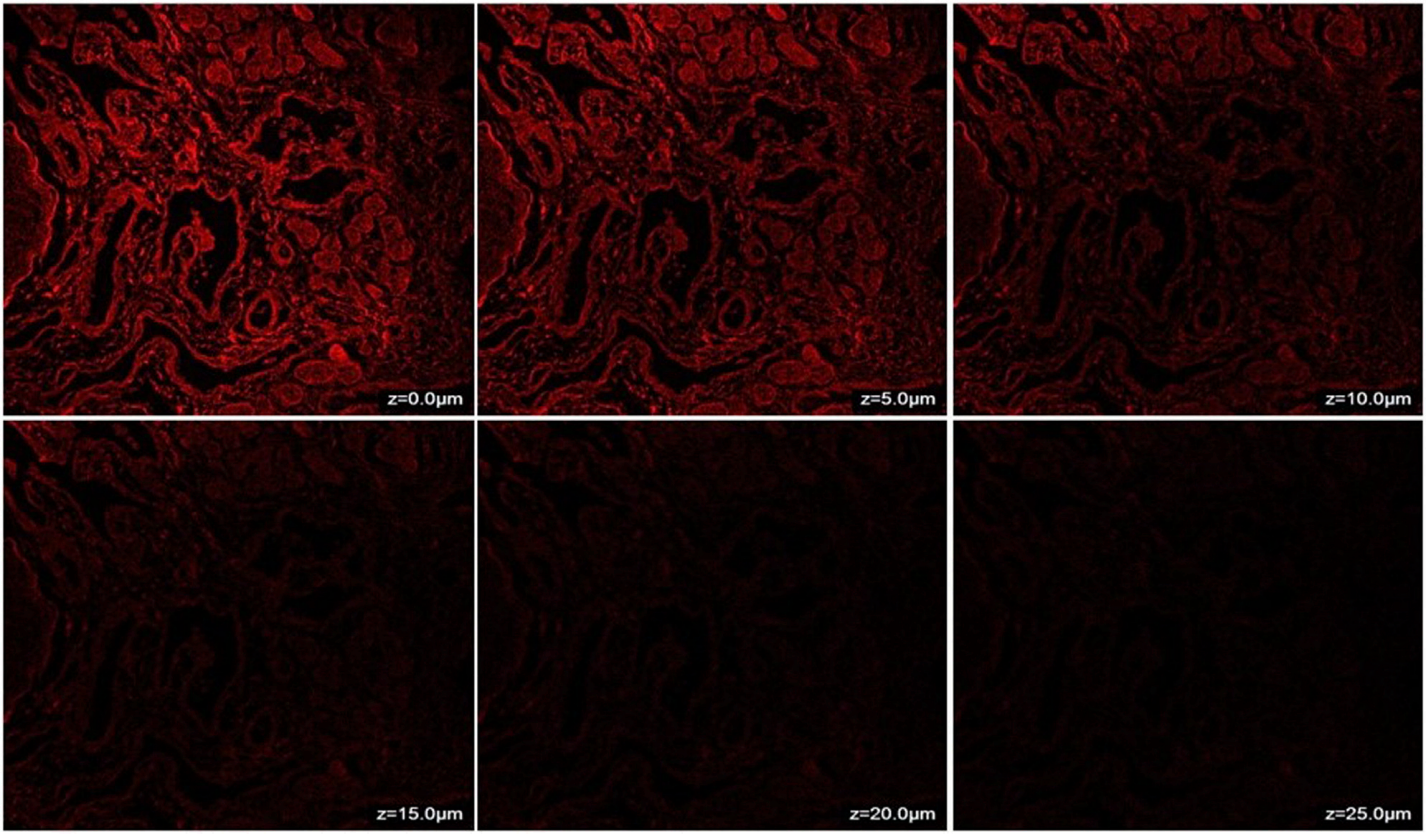
CLSM (Confocal Laser Scanning Microscopy) photomicrographs of nasal mucosa after 6 h treatment with CONPs depicting depth analysis.

The penetration of CONPs through goat nasal mucosa was evaluated *ex-vivo* using confocal microscopy. The fluorescence intensity of CONPs loaded with rhodamine dye was shown in Figure 5. The maximum intensity was observed at nasal mucosal surface at ∼0 μm, while the intensity decreased when the depth increased up to 25 μm. The deep and uniform distribution of CONPs in the nasal mucus layers was evident.

### 3.4 *Ex vivo* nasal cilio toxicity

**FIGURE 6:**
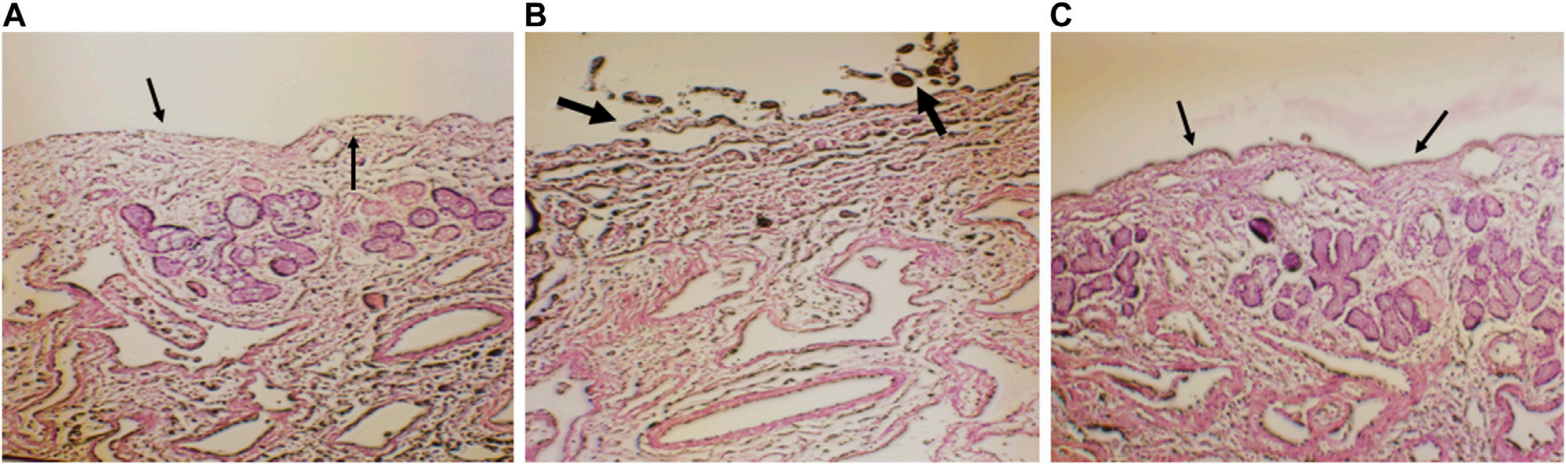
Histoptahological images of nasal goat mucosa; **(A)** negative control; **(B)** positive control and **(C)** treatment with CONPs. Thin arrows in figure **(A)** represents no nasociliary deterioration whereas, thick black arrows in figure **(B)** showed nasal mucosa was severely disrupted. Further, black thin arrows in figure **(C)** no signs of irritation or injury were observed.

Histopathological slide of negative control showed that there was no nasociliary deterioration or erosion (Figure 6A), whereas the nasal cilia were lost, and the nasal mucosa was severely disrupted with the positive control (Figure 6B). On the other hand, no signs of irritation or injury was seen with the treatment of CONPs (Figure 6C) indicating that prepared CONPs were non-irritant and safe.

### 3.5 Targeted brain delivery of intranasal CONPs by scintigraphy

**FIGURE 7:**
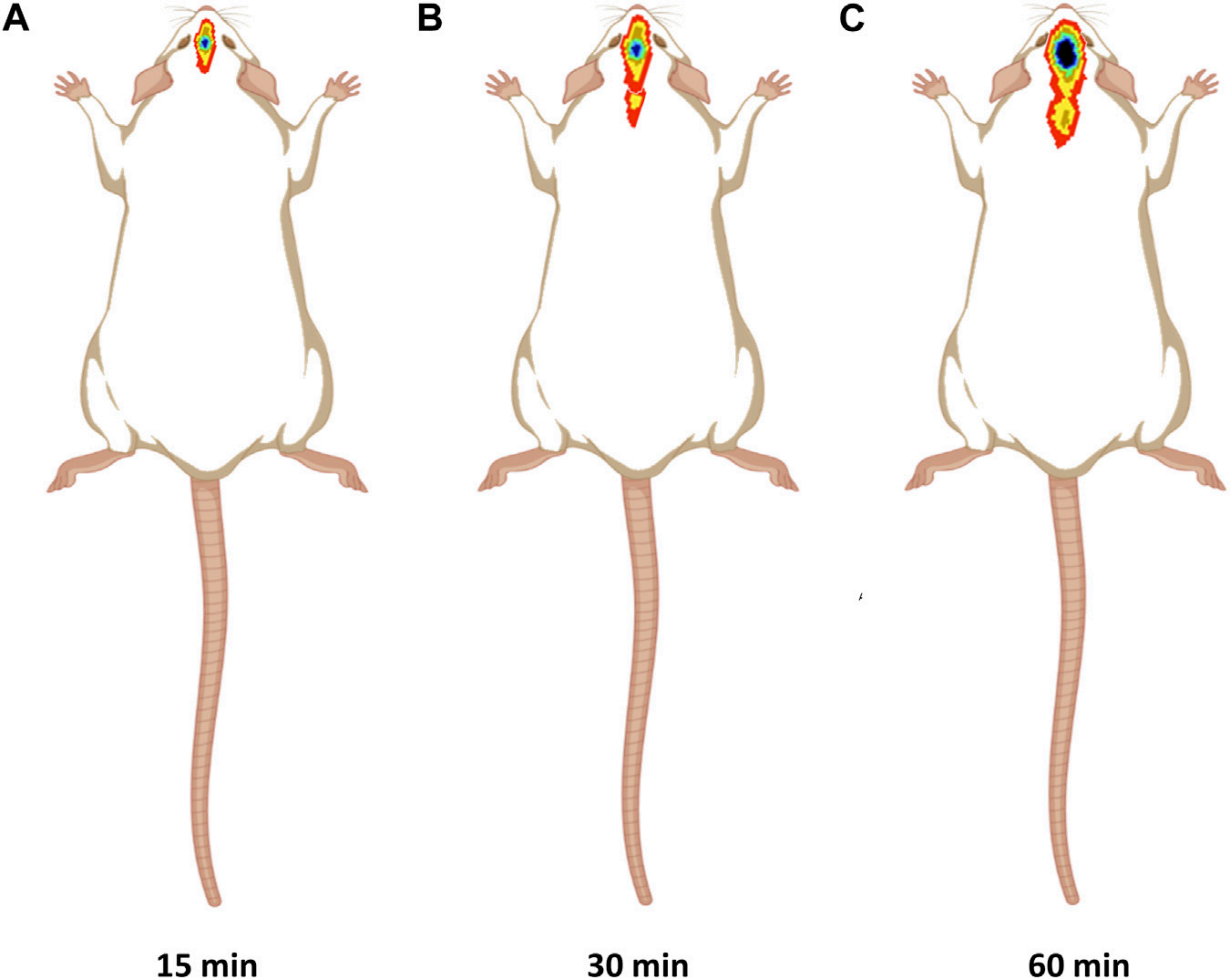
Gamma scintigraphy imaging of intranasal CONPs captured at various time intervals **(A)** 15 min, **(B)** 30 min, and **(C)** 60 min in male Sprague Dawley rats.

Figure 7A showed significant CONP accumulation in the brain at 15 min following intranasal administration. Image at 60 min revealed that the CONPs were present in the brain followed by biodistribution of small concentrations to nearby tissues as depicted by spreading of radioactivity in Figure 7C. Based on these results, more effective therapeutic impact of CONPs may be expected since radiolabeled complex had accumulated in the brains at all observed images. Our findings are consistent with the earlier report (Hasan et al., 2021).

### 3.6 Safety (acute toxicity) study

**FIGURE 8:**
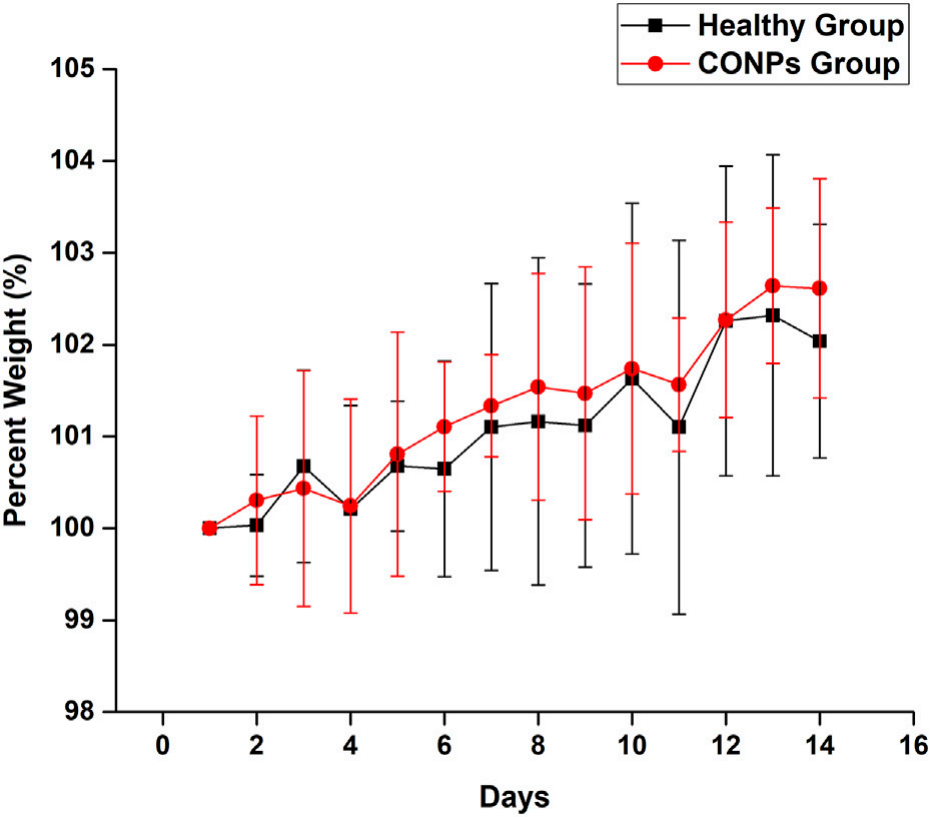
Percent weight variation in healthy group and CONPs group.

Rats having weight 250 ± 30 g were selected and maintained in a normal fed state for weight variation study to examine the changes in their body weight every day for 14 days after giving saline and CONPs. After completion of the experiment, the overall average weights of group I and group II were found to be increased by 2.14% and 2.91% respectively (Figure 8). The change in overall average weight % of group II was found to be statistically not significant (*p* > 0.05) when compared with group I. The results of the study revealed that CONPs did not cause any toxicity in experimenting animals.

### 3.7 Behavioural studies for efficacy

#### 3.7.1 Open field test

The parameters measured are: 1) Latency to move (sec); 2) Number of crossed squares; 3) Latency to rear (sec); 4) Number of rears. The results of the open field test are shown in Table 1.

**TABLE 1:** *In-vivo* Behavioural Studies Results and Effect of Developed CONPs on Different Open filed and Pole Test Parameters (*n* = 4).

	Healthy	Disease	Standard	Treatment
Open Field Parameters				
Latency to move (sec)	6.1 ± 1.38^###^	64.3 ± 3.65^***^	29.56 ± 2.21^***,###^	8.5 ± 1.50^###^
Number of crossed squares	37 ± 2.45^###^	7.5 ± 1.29^***^	22 ± 2.16^***,###^	32.75 ± 2.99^###^
Latency to rear (sec)	18.4 ± 1.41^###^	51.36 ± 2.04^***^	41.9 ± 2.98^***,##^	22.5 ± 1.11^###^
Number of rears	21.25 ± 2.98^###^	5.75 ± 1.70^***^	12.5 ± 2.38^***,##^	19.75 ± 1.5^###^
Pole Test				
t-turn (sec)	4.5 ± 1.12^###^	48.33 ± 5.03^***^	28.83 ± 2.75^***,###^	10.66 ± 3.51^###^
t-total (sec)	12.26 ± 2.58^###^	91.33 ± 6.65^***^	60.66 ± 4.04^***,###^	21.73 ± 2.15^###^

^***^ *p* < 0.001 when compared with healthy group.

^###^ *p* < 0.001when compared with disease group.

^**^ *p* < 0.01when compared with healthy group.

^##^ *p* < 0.01when compared with disease group. All data expressed as mean ± SD.

Latency to move in open field test is defined as time in seconds to leave the centre or start the movement in open field. It was found significantly (*p* < 0.001) high in the haloperidol-induced control group compared to the negative control suggestive of significant impairment in motor manifestations following administration of haloperidol. In the haloperidol-induced treated with the SD group shielding effects against haloperidol were observed as the latency to move time was significantly less than the haloperidol-induced control group (*p* < 0.001). Notably, the haloperidol-induced treated with CONPs group also exhibited latency to move time significantly (*p* < 0.001) different from haloperidol-induced control group but not significantly (*p* > 0.05) different from the negative control group.

Haloperidol-induced control group exhibited significant reduction in number of crossed squares (*p* < 0.001) compared to negative control group indicative of impairment in motor manifestations following administration of haloperidol. Haloperidol-induced treated with the SD group demonstrated significant increase (*p* < 0.001) in number of crossed squares from haloperidol-induced control group. However, haloperidol-induced treated with CONPs group showed number of crossed squares results identical to negative control group (*p* > 0.05) and significantly different (*p* < 0.001) from haloperidol-induced control group.

Administration of haloperidol significantly increased (*p* < 0.001) the latency to rear in open field test when compared to negative control group. When rats were co-treated with oral levodopa (haloperidol-induced treated with the SD group), there was significant decreased (*p* < 0.01) in latency to rear as compared to haloperidol-induced control group. Upon treatment with optimized CONPs along with oral levodopa (haloperidol-induced treated with CONPs group), significant decreased (*p* < 0.001) in the latency to rear was observed when compared to haloperidol-induced control group. However, the results are not different from negative control group (*p* > 0.05).

#### 3.7.2 Pole test

The total time to drop to the floor (T-total) and the amount of time to turn entirely downward (T-turn) are both recorded. The results of pole test are shown in Table 1.

Haloperidol-induced control group exhibited significant increase in T-turn and T-total (*p* < 0.001) compared to negative control group indicating impairment in motor manifestations and exploratory behaviour following administration of haloperidol. Haloperidol-induced treated with the SD group demonstrated significant decreased (*p* < 0.001) in T-turn and T-total from haloperidol-induced control group. However, haloperidol-induced treated with CONPs group showed T-turn and T-total results identical to negative control group (*p* > 0.05) and significantly different (*p* < 0.001) from haloperidol-induced control group.

### 3.8 Histopathological studies

**FIGURE 9:**
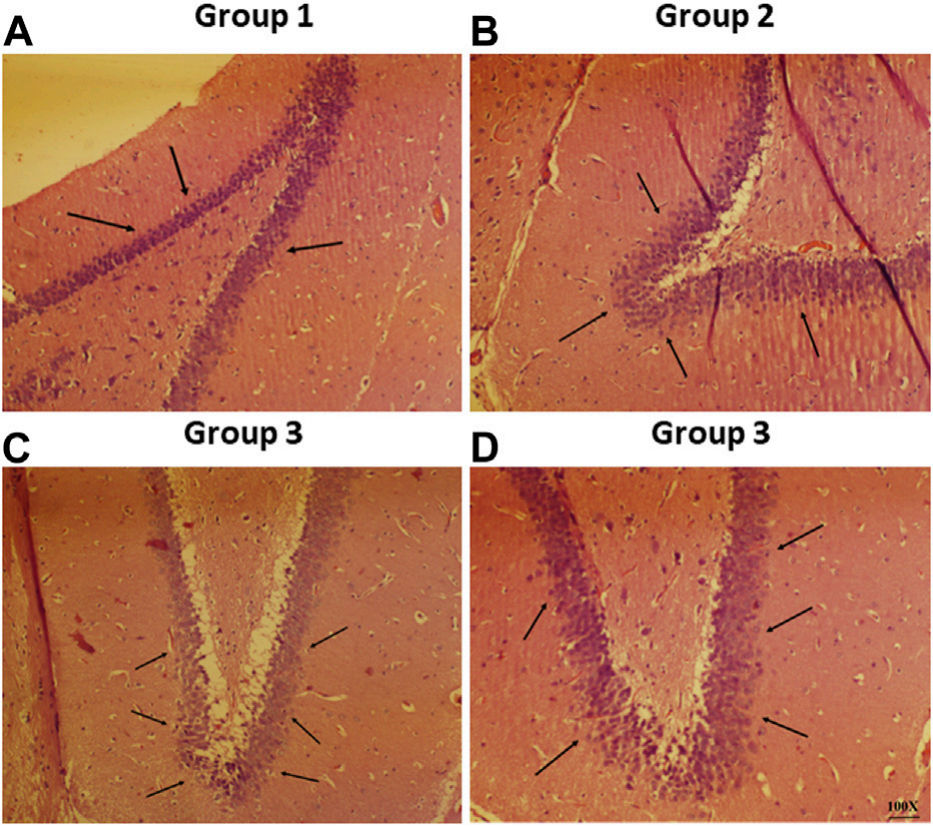
Histopathological investigation of brain samples using H and E staining observed at 100× magnification with special focus on hippocampus region whereas figure **(A–D)** represents hippocampal region of Group 1 (negative control), Group 2 (haloperidol-induced control), Group 3 (haloperidol-induced treated with the SD drug), and Group 4 (haloperidol-induced treated with CONPs) respectively. Black arrows in figure **(A)** showed no lesions whereas, arrows in figure **(B)** represents neuronal damage. Moreover, black arrows in figure **(C)** showed small and scattered living cells and in figure **(D)** congregate live cells were observed.

Representative photomicrographs of rat brain from all the study groups is shown in Figure 9. The normal pathology in the section of the CA1 region from the negative control group was observed since there were no lesions discovered in any of the neuronal cells in the shown photomicrographs (Figure 9A). On the other hand, haloperidol produced shrinkage of cellular nuclei and neuronal damage in the haloperidol-induced control group (Figure 9B). However, the neuronal damage was reduced in haloperidol-induced treated with the SD drug group but living cells were smaller and dispersed (Figure 9C). Further reduction in neurodegeneration was observed in haloperidol-induced treated with CONPs group along with more live cells which were greater in size (Figure 9D). This could be due to the intranasal delivery of CONPs directly to the brain in therapeutic concentration thereby reducing the toxic effect caused by haloperidol.

### 3.9 Biochemical estimation

#### 3.9.1 TBARS estimation

**FIGURE 10:**
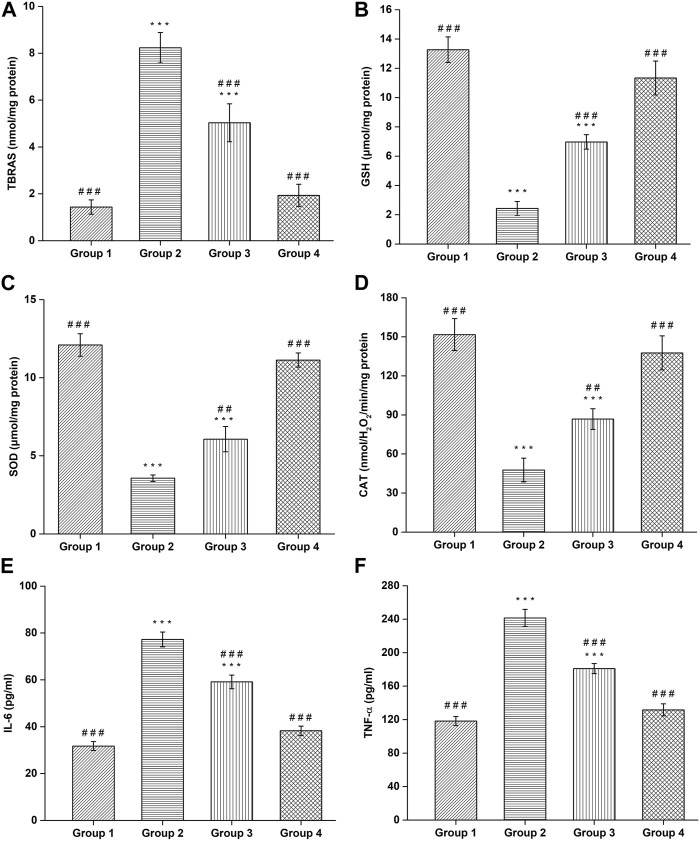
Assessment of **(A)** TBARS, **(B)** GSH, **(C)** SOD, **(D)** CAT, **(E)** IL-6, and **(F)** TNF-α. (^#^Comparison of disease group with other groups, and *comparison of healthy group with other groups). The significance was determined to be ^###^ *p* < 0.001, ^##^ *p* < 0.01 and ^#^ *p* < 0.05 respectively, vs. disease group; ***p < 0.001, **p < 0.01 and *p < 0.05 *versus* healthy group respectively.

The TBARS content was found to be 1.43 ± 0.30 nmol/mg protein in negative control group, which is significantly (*p* < 0.001) lower compared to the haloperidol-induced control group (8.23 ± 0.65 nmol/mg protein). In haloperidol-induced treated with the SD drug group, a decrease in TBARS content (5.03 ± 0.80 nmol/mg protein) was observed however it was significantly (*p* < 0.001) higher than negative control group. Interestingly, haloperidol-induced treated with CONPs group showed significant (*p* < 0.001) decrease in TBARS (1.93 ± 0.47 nmol/mg protein) compared to the haloperidol-induced control group (Figure 10A) and the values were not significantly different from (*p* > 0.05) negative control group.

#### 3.9.2 GSH estimation

Administration of Haloperidol significantly decreased (*p* < 0.001) GSH content to 2.43 ± 0.47 μmol/mg protein in haloperidol-induced control group as compared to negative control group (13.26 ± 0.87 μmol/mg protein). In haloperidol-induced treated with the SD drug group the GSH content increased to 6.96 ± 0.49 μmol/mg protein but it was significantly lower than negative control group. As expected, haloperidol-induced treated with CONPs group showed significant (*p* < 0.001) increase in the GSH content (11.33 ± 1.15 μmol/mg protein) compared to the haloperidol-induced control group and the values were not significantly different (*p* > 0.05) from negative control group (Figure 10B).

#### 3.9.3 SOD estimation

The negative control group showed SOD levels of 12.1 ± 0.72 μmol/mg protein, which is significant (*p* < 0.001) higher compared to the haloperidol-induced control group (3.56 ± 0.20 μmol/mg protein). In haloperidol-induced treated with the SD drug group an increase in SOD levels (6.06 ± 0.80 μmol/mg protein) was observed however it was significantly (*p* < 0.001) lower than negative control group. As predicted, haloperidol-induced treated with CONPs group showed significant increase (*p* < 0.001) in SOD levels (11.13 ± 0.45 μmol/mg protein) compared to the haloperidol-induced control group (Figure 10C) and the values were not significantly different from (*p* > 0.05) negative control group.

#### 3.9.4 CAT estimation

The negative control group exhibited CAT content of 151.7 ± 12.25 nmolH2O2/min/mg protein, which is significant (*p* < 0.001) higher compared to the haloperidol-induced control group (47.66 ± 9.07 nmolH2O2/min/mg protein). In haloperidol-induced treated with the SD drug group an increased in CAT content (86.83 ± 7.97 nmolH2O2/min/mg protein) was observed however it was significantly (*p* < 0.001) lower than negative control group. As expected, haloperidol-induced treated with CONPs group showed significant increase (*p* < 0.001) in CAT content (137.66 ± 13.05 nmolH2O2/min/mg protein) compared to the haloperidol-induced control group (Figure 10D) and the values were not significantly different from (*p* > 0.05) negative control group, indicating that treatment with CONPs along with levodopa showed protective antioxidant activity.

#### 3.9.5 IL-6 and TNF-α estimations

Administration of Haloperidol significantly increased IL-6 (*p* < 0.001) and TNF-α (*p* < 0.001) to 77.26 ± 3.17 pg/mL and 241.4 ± 10.19 pg/mL respectively in haloperidol-induced control group as compared to negative control group (31.73 ± 1.88 pg/mL for IL-6 and 118.26 ± 5.34 pg/mL for TNF-α). In haloperidol-induced treated with the SD drug group decreased levels of IL-6 and TNF-α level to 59.13 ± 2.91 pg/mL and 180.93 ± 6.01 pg/mL were found respectively but they were significantly higher (*p* < 0.001) than negative control group. As expected, treatment with intranasal CONPs along with oral levodopa showed significant (*p* < 0.001) decrease in the IL-6 and TNF-α level to 38.26 ± 1.97 pg/mL and 131.63 ± 7.16 pg/mL respectively compared to the haloperidol-induced control group and the values were not significantly different (*p* > 0.05) from negative control group (Figures 10E,F). CONPs suppressed the neuronal inflammatory responses by lowering the high levels of IL-6 and TNF-α and hence demonstrating the effectiveness of the produced CONPs in Parkinson’s disease.

### 3.10 Dopamine quantification

**FIGURE 11:**
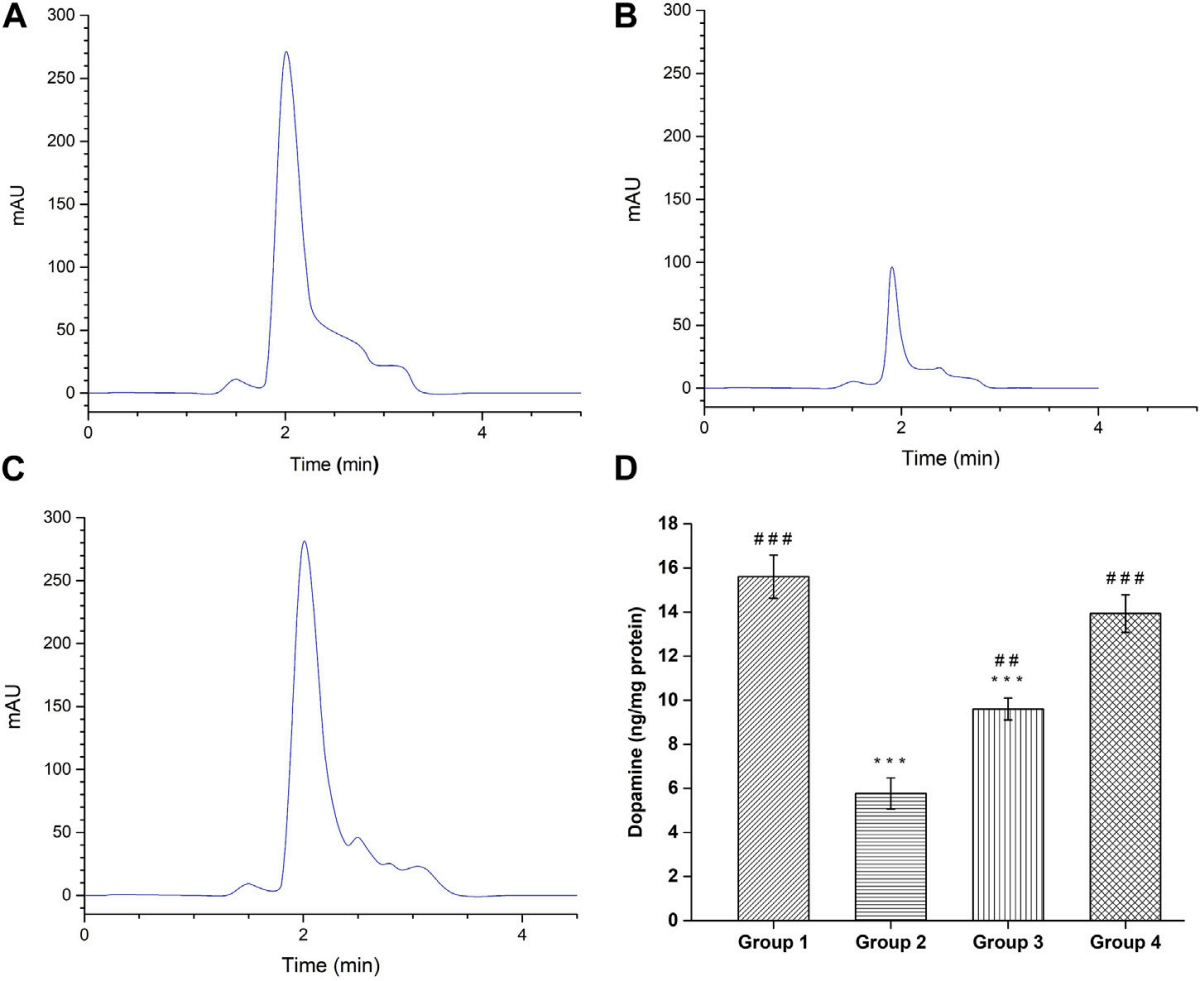
HPLC Chromatogram of dopamine by the HPLC reversed phase system. **(A)** Healthy Group; **(B)** Disease Group; **(C)** Treatment Group; and **(D)** Dopamine level in different groups. (^#^comparison of disease group with other groups, and *comparison of healthy group with other groups). The significance was determined as ^###^ *p* < 0.001, and ^##^ *p* < 0.01 *versus* disease group, respectively; ***p < 0.001 *versus* healthy group respectively.

The quantification of dopamine was done by modified RP-HPLC method and the obtained HPLC chromatograms are shown in Figures 11A–C. The negative control group demonstrated dopamine level of 15.60 ± 0.98 ng/mg protein, which is significant (*p* < 0.001) higher compared to the haloperidol-induced control group (5.76 ± 0.70 ng/mg protein). Following haloperidol-induced treated with the SD drug an increased in dopamine level (9.60 ± 0.5 ng/mg protein) was observed however it was significantly (*p* < 0.001) lower than negative control group. As anticipated, treatment with intranasal CONPs along with oral levodopa showed significant increase (*p* < 0.001) in dopamine level (13.93 ± 0.85 ng/mg protein) compared to the haloperidol-induced control group (Figure 11D) and the values were not significantly different from (*p* > 0.05) negative control group. Results showed that the treatment with CONPs along with levodopa exhibited protective activity by increasing dopamine level in treatment group.

## 4 Discussion

The pathogenesis of PD profoundly relies on oxidative stress, which lead to neuroinflammation and subsequently neurodegeneration of the brain (Hwang, 2013). The antioxidant potential of CONPs is well established and we recently reported promising effects of intranasal CONPs in the treatment of AD (Danish et al., 2022). In the present study, we assessed the therapeutic potential of intranasal CONPs in haloperidol-induced PD model. Our results showed that intranasal CONPs get accumulated in the brain and improves locomotor activity of haloperidol-induced PD in rats via suppression of oxidative stress and neuroinflammatory responses.

The role of oxidative stress in the pathogenesis of PD is well established. As a result of common cellular processes, body constantly produces free radicals and ROS (Ferreira et al., 2016), which accelerates PD via decreased glucocerebrosidase activity, mitochondrial dysfunction, accumulation of α-synuclein and other toxic or aberrant proteins in neurons (Sidransky and Lopez, 2012).

The current anti-PD modalities have potential adverse effects and search for novel and effective anti-PD drugs is ongoing. The cerium oxide at the nanoscale is widely utilized in chemical and mechanical industry owing to its strong antioxidant properties. The strong and regenerative antioxidant activity of CONPs makes them promising material for biomedical activity including treatment of oxidative stress mediated disorders. From the published reports it is evident that CONPs also promote neuron survival by activating signal transduction (D’Angelo et al., 2009), alleviating Aβ-induced cell death (Dowding et al., 2014), and decreasing protein accumulation (D’Angelo et al., 2009; Naz et al., 2017).

The CONPs utilized in this study were developed by slight modification of our previously published method of CONPs preparation (Danish et al., 2022). The CONPs developed were small-sized (105.1 ± 5.78 nm), spherical (TEM), uniform (PDI, 0.119 ± 0.006) and stable (Zeta potential, −22.7 ± 1.03 mV).

Further, intranasal route allows direct delivery to the brain through the nasal mucosa via trigeminal and olfactory pathways by passing formidable BBB (Johnson et al., 2010; Pardeshi and Belgamwar, 2013; Jeong et al., 2023).

FRAP analysis, based on estimation of amount of antioxidant needed to reduce Fe^3+^ - TPTZ complex to Fe^2+^, was done to determine *in vitro* antioxidant potential of the developed CONPs. The CONPs at concentration of 25 μg/mL demonstrated strong antioxidant activity. The redox cycle between Ce^3+^ and Ce^4+^ on the surface of CONPs was presumed to be primary cause of the antioxidant mechanism as reported previously (Zhang et al., 2014).

Since the developed CONPs were meant for intranasal delivery, the penetration and toxicity of the developed CONPs was evaluated *ex vivo* using goat nasal mucosa. Confocal microscopy has been utilized for assessment of depth of penetration of CONPs in nasal mucosa. The fluorescence intensity at all the mucosal thickness demonstrated deep and uniform distribution of CONPs in the nasal mucus layer.

Absence of pathological change and irritation during nasal cilio toxicity study demonstrated preliminary safety and biocompatibility of CONPs. Similar to our results, nasal safety of CONPs was also reported previously (Usama Ashhar et al., 2022).

Scintigraphy studies in rats was performed to demonstrate nose to brain delivery of intranasal CONPs. The CONPs were radiolabeled with technetium-99m and the gamma imaging of head portion of rat was performed. From the scintigraphic images the presence of CONPs in brain was evident. The presence of CONPs in brain at 15 min indicate rapid brain delivery of CONPs, essential for PD conditions. Further, CONPs were visible till 60 min, indicating prolong retention of the CONPs in brain following intranasal administration. The quantitative percentage of CONPs in brain was not calculated. The close proximity of nose with brain in rat model does not allows accurate quantification of radioactive counts in brain.

*In vivo* acute toxicity studies were performed to demonstrate safety of intranasal CONPs. The body weight change (%) of animals receiving intranasal CONPs for 14 days was not statistically different from those receiving intranasal saline, thus demonstrating safety of intranasal CONPs.

Administration of haloperidol causes an excess of free radicals’s production in the brain, which lowers GSH, SOD and CAT levels while raising TBARS levels. The two main causes of reduced enzyme activities are: 1) the attack of oxygen free radicals on the sulfhydryl (-SH) groups of enzymes and 2) association of enzymes with peroxidation products leads to blockage of active sites of the enzymes (Bishnoi et al., 2007). The development of the variables underlying PD is linked to a decrease in GSH levels in the brain which promotes oxidative damage due to the formation of •OH radical (Sharma et al., 2020). Loss of dopamine and neurodegeneration are both closely correlated with decreased GSH levels, which encourage mitochondrial damage (Andersen, 2004). SOD shields neuronal cells from damage by breaking harmful free radicals (Kumar et al., 2018). One of the vital antioxidant enzymes that significantly reduce oxidative stress is CAT (Zameer et al., 2020). An increase in GSH, SOD and CAT levels while decrease in TBARS content indicated that the manufactured CONPs had successfully produced an antioxidant and protective effects through the intranasal route. This could be due to CONPs potential free radical scavenging activity and its higher concentration in brain via intranasal administration. Similar observations have been reported in previous studies (Danish et al., 2022).

According to reports, PD patients have significantly higher levels of IL-6 and TNF-α in their brains (Yan et al., 2014). Therefore IL-6 and TNF- α were estimated in all groups to investigate the influence of the produced formulation on these two cytokines in the haloperidol-induced PD model. Enhanced TNF- α and IL-6 release in rat brains after administration of haloperidol served as a visual representation of the increased neuroinflammatory response seen in the current study. When CONPs were delivered intranasally, the reduced levels demonstrated its anti-inflammatory potential. The findings were consistent with earlier research in which raised levels of TNF- α and IL-6 were seen in PD patients, indicating enhanced peripheral and central inflammatory responses (Qu et al., 2023).

The elevated amounts of oxidative stress and proinflammatory cytokines also activate multiple cell signalling molecules, which results in the death of neuronal cells, which is supported by histopathological research. Haloperidol produced cellular nuclei shrinkage and neuronal damage in rats as depicted in photomicrographs. In contrast, CONPs treated group showed marked reduction in neurodegeneration since more live cells with large size was observed in CA1 region of brain indicating the neuroprotective responses. This might be due to the delivery of CONPs directly to the brain intranasally in an adequate quantity reducing the toxic effect caused by haloperidol. Gamma scintigraphy showed highest amount of CONPs accumulation in the brain after 15 min of intranasal administration which start spreading indicating bio-distribution of radiolabel-CONPs into other parts of body. Similar findings have been reported previously (Hasan et al., 2021).

Dopamine is a neurotransmitter for regulating Parkinson’s disease. The previously noted striatal dopamine depletion in PD may provide an explanation for the impairment of motor skills (Richardson and Hossain, 2013). According to the available data, haloperidol treatment drastically reduced striatal dopamine in rats. The resulting oxidative stress may be responsible for the dopamine depletion seen in the control rats. The treatment with standard drug showed increased in dopamine level however it was significantly (*p* < 0.001) lower than negative control group which could be due to the use of sub therapeutic (half) dose of oral levodopa suspension. The treatment with optimized CONPs along with levodopa significantly (*p* < 0.001) increased the level of striatal dopamine compared with haloperidol-induced control group. This could well be explained due to the regenerative free radical scavenger and enhanced antioxidant activity of CONPs (Zhou et al., 2011) and decreased dopaminergic neurons’ apoptosis (Pirmohamed et al., 2010).

*In-vivo* behavioural studies were carried out in rats using open field test and pole test. Haloperidol administration in the haloperidol-induced control group led to a significant impairment in exploratory behaviour and locomotor activity as demonstrated by a prolonged latency to start moving and rearing as well as a decreased number of crossed squares and number of rears compared with negative control rats, which suggests bradykinesia in this group. Rats treated with sub therapeutic (half) dose of oral levodopa suspension showed protective effects and all the parameters were significantly different (*p* < 0.001) from disease group however the parameters were not similar to negative control group. As expected, rats treated with 6 mg/kg of CONPs with sub therapeutic (half) dose of oral levodopa suspension exhibited significant increase (*p* < 0.001) in the number of crossed squares and rears, as well as a significant decrease (*p* < 0.001) in latency to move and rear, indicate protective effects of CONPs. Similar results were observed in pole test. Furthermore, all the parameters in both tests were not significantly different from (*p* > 0.05) negative control group. This could be explained by the existence of CONPs in the treatment group and by the fact that they have the capacity for regeneration and redox processes, which contribute to their long-lasting effects as previously documented. (Szymanski et al., 2015; Dhall and Self, 2018). The results of all studies demonstrated improved motor manifestations and neurochemical impairments in haloperidol induced PD.

## 5 Conclusion

Oxidative stress is thought to have a significant role in the pathogenesis of Parkinson’s disease. The effectiveness of intranasal CONPs was assessed utilizing antioxidant activity, biochemical estimations and *in vivo* behavioral studies using a haloperidol-induced PD model. Nasal cilio toxicity and histopathological studies revealed its safety and biocompatibility with surrounding tissues. Biochemical assessment studies were done to estimate the levels of SOD, GSH, CAT, and TBARS in all animal groups. Along with oxidative stress measurements, levels of neuroinflammation cytokines like IL-6 and TNF-α were also estimated. The amount of thiobarbituric acid reactive substances (TBARS) was reduced significantly, whereas the levels of catalase (CAT), superoxide dismutase (SOD), and GSH were increased significantly, while amounts of interleukin-6 (IL-6) and tumour necrosis factor-alpha (TNF-α) showed significant reduction after intranasal administration of CONPs. Treatment with intranasal CONPs along with oral levodopa showed significant increase (*p* < 0.001) in dopamine level compared to the disease group. In efficacy evaluation using the open field and pole test, the intranasal CONPs produced protective effect against haloperidol induced PD. In treatment group, all parameters were found to be significantly different (*p* < 0.001) from disease group. The overall results demonstrated that intranasal CONPs markedly improved neurochemical impairments and motor manifestations in rats and hence, showed the effectiveness of intranasal administration of CONPs in ameliorating oxidative stress against haloperidol induced PD. Intranasal delivery of CONPs can be considered as a future prospect in potential management of PD owing to its antioxidant activity.

## Data Availability

The original contributions presented in the study are included in the article/Supplementary Material, further inquiries can be directed to the corresponding authors.
